# Automatic LV localization and view planning for cardiac MRI acquisition

**DOI:** 10.1186/1532-429X-13-S1-P39

**Published:** 2011-02-02

**Authors:** Peter Kellman, Xiaoguang Lu, Marie-Pierre Jolly, Xiaoming Bi, Randall Kroeker, Michaela Schmidt, Peter Speier, Carmel Hayes, Jens Guehring, Edgar Mueller

**Affiliations:** 1 National Institutes of Health, Bethesda, MD, USA; 2 Siemens Corporate Research, Princeton, NJ, USA; 3 Siemens Medical Solutions USA, Chicago, IL, USA; 4 Siemens Medical Solutions, Canada, Winnipeg, MB, Canada; 5 Siemens AG, Healthcare Sector, Erlangen, Germany

## Background

Localization of the heart is typically performed using a multi-step approach involving the acquisition of double-oblique localizer images. Based on the localizers the standard heart views are planned. This approach is operator-dependent and time consuming.

## Objective

To demonstrate feasibility of a fully automatic and fast approach to heart localization and slice prescription from a highly-accelerated, single breath-hold 3D acquisition through a machine learning method.

## Methods

A 3D full-chest MR scan is obtained through parallel imaging within a single breath-hold. A single volume is acquired at mid-diastole using an ECG gated segmented acquisition with T2-prepared SSFP readout with chemical shift fat suppression. Typical protocol parameters are: 400x400x220 mm^3^ FOV prescribed as a coronal slab (256x202x44 matrix; 1.6x2x5 mm^3^ resolution, interpolated to 2.5 mm slices). Images are acquired using a 1.5T Siemens Avanto/Espree with 32 channel coil and parallel imaging with rate 6=3x2 (PE in LR direction x PAR in AP direction) and ¾ partial Fourier in PAR dimension. Breath-hold duration is typically less than 20s with all PE lines acquired in a single shot per heartbeat.

**Figure 1:**
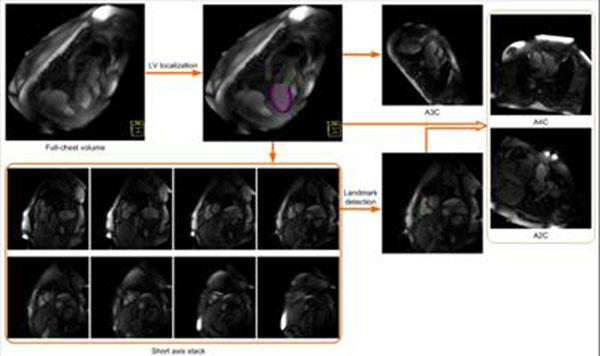
Workflow of automatic LV localization and view planning

We train a series of detectors on a database with manually delineated LV to estimate the LV pose and boundaries using probabilistic boosting trees [1] and marginal space learning [2]. The short axis stack is planned based on the delineated LV. To prescribe the VLA and HLA views, we choose a mid-ventricular short axis slice, and apply a landmark detection algorithm [3] to localize the RV insertion points, and the RV lateral point (Figure 1). The VLA view is calculated to be parallel to the line connecting the RV insertion points and to cross the LV blood pool center. The HLA view is computed to cross the LV blood pool center and the RV lateral point.

## Results

We collected 49 MR volumes and manually delineated LV on 27 volumes. We apply our method and visually inspect the results. For cases with full LV coverage, our method delineates LV sufficiently accurate for planning purposes (mean error: 2.10mm, standard deviation: 0.98mm). Failures are mostly due to partial LV coverage.

## Conclusion

We successfully demonstrated feasibility of a fully automatic and fast approach to heart localization and slice prescription from a single breath-hold 3D scan in less than 30 seconds. Further investigations to characterize the performance and robustness of the method are warranted.

**Figure 2:**
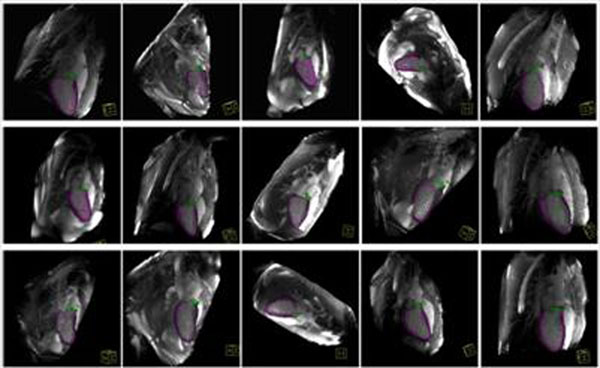
Examples of automatically localized LV
